# A re-usable wave bioreactor for protein production in insect cells

**DOI:** 10.1016/j.mex.2016.08.001

**Published:** 2016-08-05

**Authors:** J. Scholz, S. Suppmann

**Affiliations:** Biochemistry Core Facility, Max-Planck-Institute of Biochemistry, Martinsried, Germany

**Keywords:** A re-usable wave bioreactor for protein production in insect cells, Insect cells, Sf9, Hi5, Upscale, Bioreactor, Wave bag, Protein production

## Abstract

Wave-mixed bioreactors have increasingly replaced stainless steel stirred tank reactors in seed inoculum productions and mammalian cell-based process developments. Pre-sterilized, single-use plastic bags are used for cultivation, eliminating the risk of cross-contamination and cleaning procedures. However, these advantages come with high consumable costs which is the main barrier to more uptakes of the technology by academic institutions. As an academic Core Facility that faces high demand in protein production from insect cells, we have therefore developed a cost-effective alternative to disposable wave bags. In our study we identified:

• A re-usable wave shaken polycarbonate bioreactor for protein production in insect cells achieves protein yields comparable to disposable bags.
• The advantages of this re-usable bioreactor are low costs, long life cycle, flexible configuration of accessories and convenient handling due to its rigid shape.

## Graphical abstract

**Figure:**
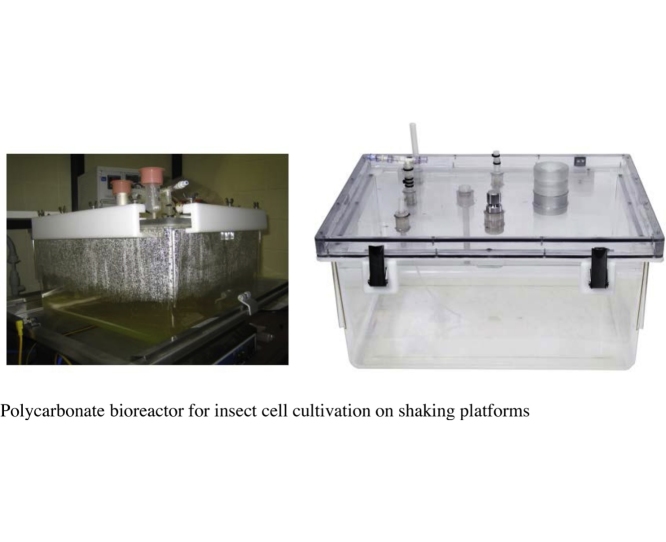

## Method details

### Preparation of bioreactor

1. As a cultivation container, a polycarbonate box 500 U Eurostandard Typ IV S, TECNIPLAST, Italy, Size 480 × 375 × 210 mm, Volume 38 L, originally made for animal housing was used. The material is transparent and autoclavable repeatedly at 121 °C. The 1st Cooper model was autoclaved and used 35 times without loss of performance.
2. The lid was constructed in-house. It consists of polycarbonate at corresponding size and is sealed with a layer of rubber foam. Six metal clamps on four sides guarantee tight attachment to the base container.
3. Within the lid, seven ports with connectors to both inner and outer sides are inserted. These insertions are screw threads manufactured in-house.
  a. Two openings are standard 13.5 Pg ports for optional pH or pO_2_ sensors (not used in our settings).
  b. One single wide-mouth opening of 5.4 cm inner diameter serves as air outlet, covered with C55 Silicon Cap (Hirschmann, VWR, Cat No 8905755) and protected by aluminium foil during autoclaving.
    Another four ports (one wildcard) at inner diameter of 0.5 cm have been introduced for
  c. Air inlet, sterile filtered through an Acro 50, 0.2 μm PTFE filter, (Pall, P/N 4151), also covered by aluminium foil for autoclaving.
  d. Aseptic sampling with Super Safe Sampler (Infors, Cat No 65373) and thin inside tubing (ID 2 mm, OD 4 mm), that allows to take smallest sample volumes without any risk of introducing contaminations.
  e. Medium fill via a long (outside 2.30 m, inside 24 cm) tubing, connected via stainless steel quick connectors (Stäubli, Cat No RBE06.1806/IC/OD/JE, RBE06.7806/IC/JE) to a 1 L Schott bottle, pressure balanced by an Acrodisc CR 0,45 μm PTFE sterile filter (Pall) for autoclaving.
4. Pharma-compliant platinum cured silicone tubings (Applied Critical Fluids, Cat No I-SA P60 40-24-88, ID 4.0, OD 8.8; Wall 2.4 mm) were used. The tubings are suitable for peristaltic pumps and autoclavable.
5. Bioreactor and connected Schott Bottle are sterilized by autoclaving (121 °C, 15 Ψ, 20 min). After sterilization, the Schott bottle can be replaced by the medium reservoir or starting culture eg. in a 2 L bottle to be pumped into the reactor. This port can also be used for fed-batch during cultivation.

### Small and large scale insect cell culture protocols

1. Sf9 (Life Technologies) or Hi5 cells (provided by Gene Center, LMU Munich) were grown in suspension in Ex-Cell 420 (Sigma, Cat No 24420C). Cell count, viability and cell diameter were analyzed using the Vi-cell XR cell counter (Beckman Coulter). Cells were maintained at a density 1–5 × 10^6^ cells/mL and 0.2–5 × 10^6^ cells/mL for Sf9 and Hi5 cells respectively. Cell diameter is about 19 μm, and viability should be higher than 95%. It is important to ensure that cells are approximately those values in order to maintain high quality and reproducibility of experiments.
2. Cells were grown in shaker incubators (Infors, 50 mm rotating diameter) at 27 °C in Erlenmeyer EM or Fernbach FB glass flasks covered with Silicon Caps (Hirschmann, VWR) at the following combinations of culture volume − flask volume − shaking speed: 30 mL − 250 mL EM − 120 rpm; 150 mL − 1 L EM − 120 rpm; 200 to 400 mL − 1,8L FB − 90 rpm; 400 mL to 1 L − 5 L EM − 90 rpm
3. Baculovirus expression was performed according to the Bac-to-Bac^®^ protocol (Life Technologies). Bacmid transfections in Sf9 cells were harvested after 3–5 days. Virus titer was determined using the SF9-ET easy titer cell line [1]. Virus was either amplified in two subsequent steps to generate Passage 1 and Passage 2 virus stocks or used to generate Baculo Infected Insect Cells (BIIC) as described previously [2]. BIICs frozen at −80 °C served both for protein expression and as virus storage. For protein expression, Hi5 and SF9 cells were adjusted to 1 × 10^6^ cells/mL and infected with virus stock or BIICs at different dilutions, typically in the range 1:1000–1:10,000.

## Method validation

**Fig. 1:**
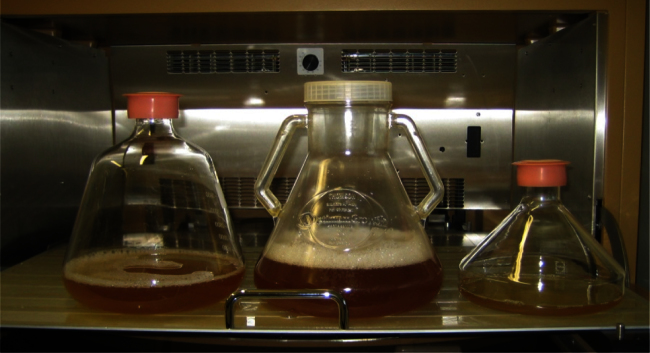
Protein production in 5 L Erlenmeyer, Thomson Flasks and Fernbach Flasks.

For protein production at 1 L scale we are using 5 L Erlenmeyer glass flasks (modified in shape, see Fig. 1) and have also tested Thomson Optimum Growth Flasks (Cat No 931116) filled with 2 L culture volume. In order to evaluate the upscale to 5 and 10 L, protein expression was first compared between flasks, Wave disposable Bags (GE Healthcare, Cat No CB0010L10-01), and the newly developed bioreactor, named Cooper. Both containers were shaken on a Wave System 20/50 EH. Parameters previously optimized at small scale were: temperature 26 °C; production time 72 h; cell density at infection 1 × 10^6^ cells/mL. The optimal rocking rates and angles were optimized individually for both bioreactors based on cell viability and productivity of a target protein Zyx-LIM-eGFP. For Wave Bags, a rocking rate and angle of 28 rpm and 9° respectively, were recommended by the manufacturer and resulted in best productivity (Table 1). For the Cooper bioreactor, the rocking rate and angle were adapted to achieve a fluid wave comparable to the Wave Bag as well as maximal cell viability and Zyx-LIM-eGFP productivity. The resulting parameters were 30 rpm and 7,5°. In summary, productivity of Zyx-LIM-eGFP was comparable in all cultivation containers tested as shown in Table 1.

**Fig. 2:**
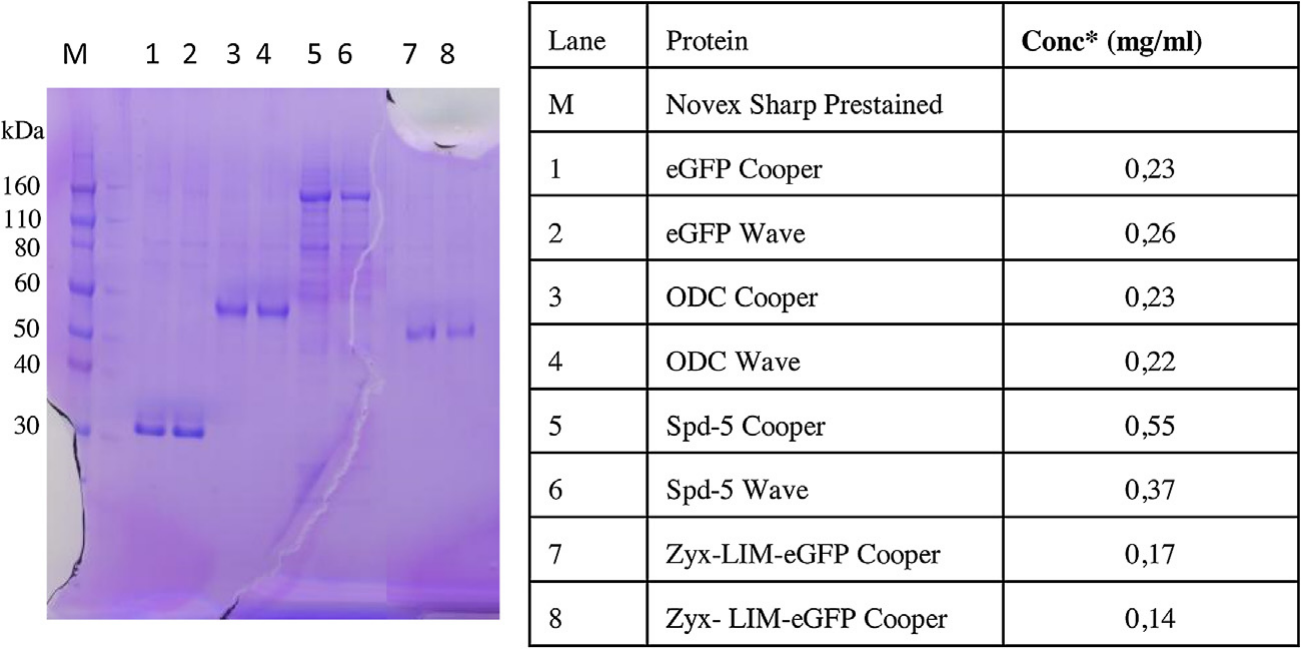
Coomassie stained SDS-PAGE of proteins produced in Wave and Cooper bioreactors and purified by One-Step Immobilized Affinity Chromatography. Protein concentration was measured by Bradford staining.

Encouraged by the results, productivity at 5 L scale in Wave Bags versus Cooper Bioreactor for three more proteins was compared next. Protein yield, concentration and purity in the re-usable bioreactor were comparable in Cooper and Wave Bags (Table 2 and Fig. 2). Comparisons of cell parameters at harvest show higher cell viability in three out of four Wave Bag productions. Despite having omitted any antifoam reagent in the ExCell 420 medium, no foaming was observed in both bioreactors, as illustrated in the Graphical Abstract showing Zyx-LIM-eGFP production in the Cooper bioreactor.

## Additional information

Space is rapidly becoming limited when scaling up insect cell suspension cultures for production. One Infors shaker can accommodate 6 × 5 L Erlenmeyers flasks, giving 6 L production capacity. There are different solutions to that space limitation, the choice of which depends very much on the individual equipment available. Most common are bioreactors especially in the pharmaceutical industry, albeit less represented in academic laboratories. In the past few years, alternative choices have emerged, like high cell density protocols [4] or culture vessels that can be filled up to 5 L [5]. Having Labfors stirred-tank bioreactors and Wave Platforms available, we invested efforts in bioreactor cultivation.

Upscale to 5 L was first performed in a stirred-tank bioreactor (Labfors, Infors, 7.5 L) adapted to insect-cell cultivation with integrated pH and pO_2_ measurement and regulation. Expression levels were comparable to flask expression (data not shown). Most interestingly was the fact that pH and pO_2_ was very stable throughout the entire cultivation and protein production process and did not need any correction. As a consequence, the rather labor-intensive setup of the Labfors stirred-tank bioreactor was discontinued and replaced by Wave Bags. The advantage of Wave-mixed agitation is the efficient nutrient distribution, off-bottom cell suspension and efficient oxygen transfer without the damaging shear forces induced by mechanical stirring and gas sparging in stirred-tank bioreactors [6]. There are a variety of systems commercially available, that differ in bag design, sensor types and type of platform movement (reviewed in [7]). The mixing is driven by the oscillating movement of a platform and the mixing rate is controlled by modifying the rocking rate, rocking angle, filling volume and aeration rate of the shaken bag. With the development of a re-usable Wave-shaken bioreactor^1^ that can be operated on these platforms, we exploited all these advantages without increasing our running costs for consumables. Apart from that cost reduction, some more advantages of our re-usable Wave Bioreactor were identified during the evaluation process:

1. Despite a filter heating integrated in the Wave system, the air outlet filter of a Wave Bag was occasionally wetted by condensing water in the tubing and as a consequence, air transfer was blocked. To circumvent this problem a wide-mouth port covered by a Silicon cap was instead inserted as air outlet. This guaranteed undisturbed air circulation and also assured sterility.
2. The filling-volume of the box is very flexible in the range of 2–10 L
3. A disposable Wave Bag obtains its shape by filling it with air and the shape is maintained by constant air circulation. In contrast, the re-usable polycarbonate reactor has a rigid and robust shape which is insensitive to errors in air exchange like filter wetting.
4. The geometric plane shape together with the grip-like lid facilitates handling especially in transport.

In summary, this new re-usable bioreactor is most suitable for academic institutions and productions of low-value products, whereas for high value products, single-use bioreactors will still be preferred. At the Biochemistry Core Facility of MPIB, this bioreactor enabled the cost-effective upscale of protein production in insect cells to meet the increasing needs of the users. Todate we have performed fifty Cooper productions at 2–10 L scale, among them Spd-5 [3] and ATG1 [8], with a contamination rate of 0,5% and 35 life cycles of the 1st generation Cooper bioreactor.

## Figures and Tables

**Table 1:** Productivity in different culturing containers.

Container type	Container Volume	Culture Volume	Yield mg/L
Fernbach Glass	1.8 L	0.5 L	29
Fernbach Glass	2.8 L	0.8 L	21
Erlenmeyer Glass	5 L	1 L	24
Thomson Optimum Flask	5 L	2 L	25
Wave Bag	10 L	5 L	21
Cooper	38 L	5 L	26

His-mZyx-LIM-eGFP (Lim domain from mouse Zyxin, Uniprot Q62523, fused to eGFP;) was produced for 72 h in Hi5 cells. Protein was purified by one-step immobilized metal affinity chromatography.

**Table 2:** Comparison of protein production in Wave Bag versus Cooper.

Protein	Yield (mg/L)	Cells	Viability at infection (%)	Diameter at infection (μm)	Viability at harvest (%)	Diameter at harvest (μm)
eGFP Cooper	34	Hi5	96	19	75	25
eGFP Wave	39	Hi5	96	20	84	25
ODC1Cooper	34	Hi5	94	20	70	25
ODC1 Wave	33	Hi5	97	20	77	26
Spd-5 Cooper	83	Sf9	97	21	80	27
Spd-5 Wave	56	Sf9	97	21	87	29
Zyx-LIM-eGFP Cooper	26	Hi5	93	21	78	27
Zyx-LIM-eGFP Wave	21	Hi5	93	21	68	26

Cells were infected at 1 × 10^6^ cells/mL. Proteins were produced at 5 L scale for 72 h. All proteins were His tagged and purified by one-step immobilized affinity chromatography. ODC1 (Homo sapiens Ornithindecarboxylase, Uniprot P11926; modified); Spd-5 ([3], *C. elegans*, Uniprot P91349).

## Footnotes

1 The Cooper Bioreactor is subject of a Utility Model Protection No 2 398 487-0001.
